# "Cutting Through the Storm": Surgical Rescue of an Electrical Storm in Post-Myocarditic Aneurysm

**DOI:** 10.7759/cureus.87405

**Published:** 2025-07-07

**Authors:** Dorian du Bus de Warnaffe, Matteo Pettinari, Maria-Chiara Badii

**Affiliations:** 1 Cardiology, Cliniques Universitaires Saint-Luc, Brussels, BEL; 2 Cardiac Surgery, Cliniques Universitaires Saint-Luc, Brussels, BEL

**Keywords:** cryoablation, dor procedure, va-ecmo, venoarterial extracorporeal membrane oxygenation (va-ecmo), ventricular aneurysm, ventricular tachycardia

## Abstract

Ventricular tachycardia (VT) refractory to medical therapy and catheter ablation remains a challenging condition, particularly when associated with localized ventricular aneurysms. Surgical ventricular reconstruction combined with intraoperative cryoablation is rarely performed but may be curative in selected cases.

We present the case of a 43-year-old man with recurrent VT originating from a localized inferobasal aneurysm secondary to prior myocarditis. Despite four endocardial and one epicardial catheter ablations performed in multiple centers, optimal medical therapy, and subcutaneous implantable cardioverter-defibrillator (S-ICD) implantation, the patient experienced electrical storm requiring extracorporeal membrane oxygenation (ECMO) and continuous venovenous hemodiafiltration (CVVH). After multidisciplinary discussion, surgery was decided due to previous ablation failures and a well-identified arrhythmogenic substrate.

Surgical ventricular reconstruction using the Dor procedure and intraoperative cryoablation was performed following careful multidisciplinary evaluation. The aneurysmal tissue was successfully resected, and the left ventricle was reconstructed with a tailored Dacron patch. Cryoablation targeted arrhythmogenic zones around the resection margins. The patient recovered uneventfully, was weaned off ECMO and CVVH within 24 hours, and remained asymptomatic without VT recurrence during an 11-month follow-up.

This case highlights the potential role of surgical ventricular reconstruction with cryoablation in managing refractory VT due to post-myocarditic ventricular aneurysm within a multidisciplinary framework.

## Introduction

Electrical storm, defined as the occurrence of multiple episodes of ventricular tachycardia (VT) or ventricular fibrillation within a 24-hour period, represents a major cardiac emergency associated with high morbidity and mortality, particularly in patients with underlying structural heart disease [1,2]. This clinical scenario exposes patients to a significant risk of sudden cardiac death and requires rapid, multidisciplinary management.

In patients with a left ventricular aneurysm following myocardial infarction or myocarditis, the arrhythmogenic substrate is typically located at the junction between scar tissue and viable myocardium. This anatomical configuration often makes it difficult to control ventricular arrhythmias using medical therapy or percutaneous catheter ablation alone [3-5].

Surgical ventricular reconstruction, as described in the Dor procedure, aims to restore ventricular geometry and exclude aneurysmal tissue. This approach also provides direct access to the arrhythmogenic substrate, allowing targeted surgical ablation [3,4]. When combined with intraoperative cryoablation, applied at the interface between scar and viable myocardium, this strategy has demonstrated notable efficacy in preventing VT recurrence, with long-term success rates exceeding 85% in certain series [3,4,6].

Leading scientific societies endorse this combined surgical and ablative approach as a validated therapeutic option for patients with deep intramural circuits or difficult epicardial access, particularly when catheter ablation has failed or is not feasible [5,7,8].

However, the Dor procedure is not routinely employed for post-myocarditic ventricular aneurysms, unlike post-ischemic aneurysms; its use in this context is limited to exceptional cases reported in the literature [9]. Left ventricular aneurysms secondary to myocarditis exhibit several notable differences compared to post-infarction aneurysms, spanning clinical presentation, pathophysiology, and management.

Clinically, post-myocarditic aneurysms occur in younger patients, often lacking traditional cardiovascular risk factors, and may manifest with severe ventricular arrhythmias, sometimes more frequently than post-ischemic aneurysms. Furthermore, post-myocarditic aneurysms more commonly involve basal segments, whereas post-infarction aneurysms are typically apical or anterior [10,11].

Pathophysiologically, post-infarction aneurysms result from transmural necrosis followed by fibrotic scarring, while post-myocarditic aneurysms stem from focal or diffuse inflammation, with significant variability depending on etiology. The aneurysmal wall in myocarditis may be less thinned and less well-demarcated than in ischemia, complicating surgical indication and reconstruction [8,10,12,13].

From a management standpoint, the Dor procedure is well-established for post-ischemic aneurysms with clear indications, whereas the literature reports only isolated cases of post-myocarditic aneurysms, without formal surgical guidelines.

Percutaneous ablation in post-myocarditic aneurysms is further complicated by several anatomo-pathological features. First, the arrhythmogenic substrate is often epicardial or endo-epicardial, unlike post-infarction aneurysms, where it is mostly endocardial and accessible via endocavitary approaches [10,12,13]. Second, the scar distribution in myocarditis tends to be diffuse, patchy, and localized in basal and lateral segments, with a predominance of epicardial or intramural scars, rendering electroanatomical mapping and circuit delineation more complex. Third, the depth of the substrate in myocarditis may limit radiofrequency ablation efficacy, as energy delivered solely through endocardial or epicardial routes may fail to reach the full thickness of arrhythmogenic tissue [8,13].

This clinical case is rare and adds to the few previously described cases in the literature, underscoring the challenges and potential benefits of a tailored surgical approach in this complex setting.

We report the case of a 43-year-old patient admitted for refractory electrical storm in the setting of a post-myocarditic left ventricular aneurysm, successfully managed with surgical aneurysmectomy combined with endocavitary cryoablation, illustrating the value of an integrated, multidisciplinary approach.

## Case presentation

In 2003, a 22-year-old man with no personal or family medical history, no home medication, and no symptoms underwent a routine cardiology check-up as part of military service requirements. Clinical examination and blood tests were unremarkable. A transthoracic echocardiogram unexpectedly revealed an inferobasal left ventricular aneurysm with preserved systolic function. At the time, given the patient’s young age, complete lack of symptoms, and normal physical examination and laboratory data, no further investigations or follow-ups were planned.

Between 2003 and 2012, the patient remained completely asymptomatic, with no cardiac consultations or imaging. There was no evidence of disease progression during this period.

**Figure 1:**
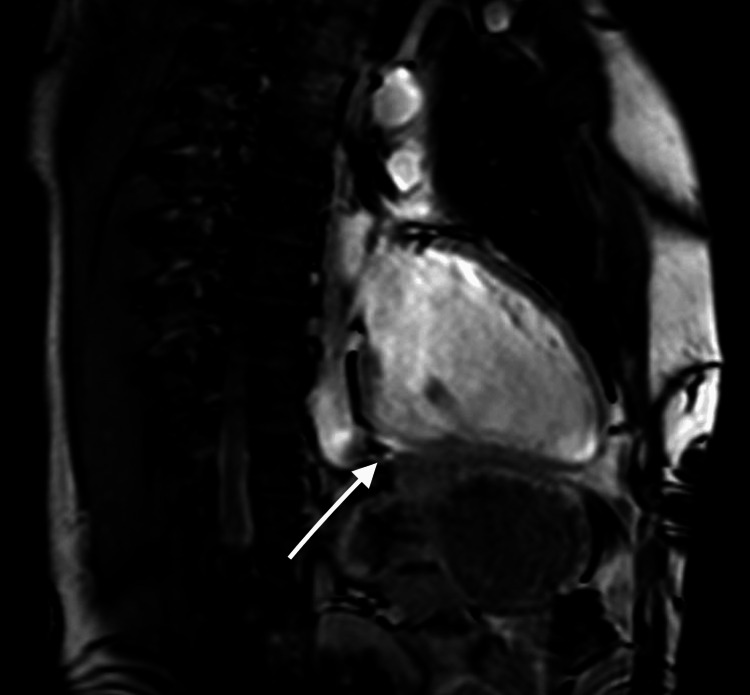
Cardiac MRI demonstrating a localized left ventricular aneurysm at the inferobasal wall. Sagittal cardiac MRI sequence showing a well-defined aneurysm of the inferobasal segment of the left ventricle. Late gadolinium enhancement indicates myocardial fibrosis consistent with a post-myocarditis sequela, which forms the substrate of the aneurysm.

**Figure 2:**
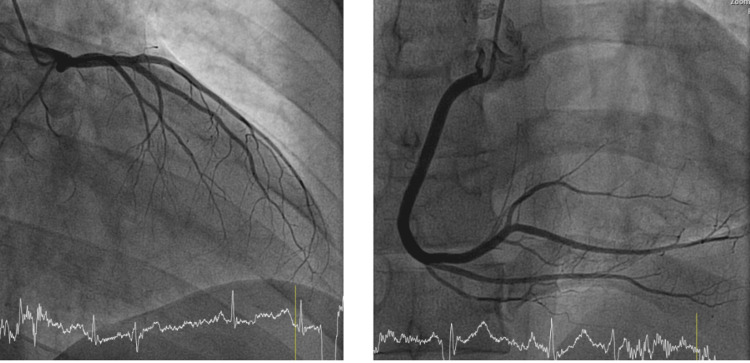
Coronary angiography demonstrating angiographically normal coronary arteries. Two angiographic images are shown: one depicting the left coronary artery system and the other depicting the right coronary artery system. Both coronary networks appear normal, without any evidence of stenosis or atherosclerotic lesions.

In 2012, at age 31, he developed palpitations and several lipothymic episodes over weeks. These episodes were self-limiting but prompted evaluation. A 24-hour Holter recorded rare, non-sustained VT. A comprehensive workup was performed. Cardiac magnetic resonance (CMR) imaging revealed a well-demarcated scar with late gadolinium enhancement and mild surrounding myocardial edema consistent with a post-myocarditic origin of the aneurysm (Figure 1). Coronary angiography showed normal, unobstructed coronary arteries without stenosis (Figure 2). Alternative diagnoses, including sarcoidosis and amyloidosis, were excluded through laboratory testing and imaging. Serial echocardiograms from 2012 onward showed stable aneurysm dimensions, no significant remodeling, and preserved left ventricular systolic function (ejection fraction [EF] consistently 50-55%). 

From 2012 to 2014, the patient experienced infrequent, symptomatic but hemodynamically tolerated episodes of sustained monomorphic VT. He was initially managed with beta-blockers (atenolol titrated to the maximum tolerated dose), but recurrences led to the addition of amiodarone. Despite this, VT episodes persisted, motivating referral for ablation in 2014.

**Figure 3:**
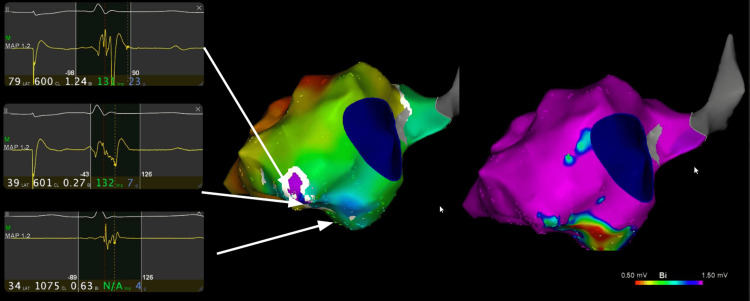
Electroanatomical mapping showing the arrhythmogenic substrate. An aneurysm was found posteroseptal and posterobasal with a zone of late potentials with later activation towards the lateral wall.

Between 2014 and 2023, the patient underwent four percutaneous catheter ablation procedures at specialized centers (Figure 3). The first three (2014, 2022, and 2023) used endocardial mapping and radiofrequency ablation targeting mid-inferobasal scar-related circuits. Despite initial success, VT recurred within months. A fourth procedure in 2023 included combined endocardial and epicardial access, targeting perivalvular and inferobasal epicardial scar regions with extensive mapping and ablation of late potentials and fractionated electrograms. Nevertheless, deep intramural circuits were suspected, making complete substrate elimination unachievable.

Pharmacological therapy over this period included amiodarone and trials of sotalol and bisoprolol. None achieved complete suppression of VT.

In 2018, given the persistence of sustained VT, a subcutaneous implantable cardioverter-defibrillator (S-ICD) was implanted for secondary prevention. Between 2018 and 2023, the S-ICD delivered three appropriate therapeutic shocks (one in 2022 and two in 2023 for sustained VT episodes without hemodynamic compromise). Otherwise, arrhythmias during this period were infrequent, with one to two episodes per month on average, mostly self-limiting.

Coronary artery disease progression was systematically excluded during follow-up. Coronary angiographies were repeated in 2018 and 2023, confirming normal coronary anatomy without stenosis, and no revascularization procedures were required.

Serial echocardiograms over the entire follow-up demonstrated stable aneurysm size, no progression of global ventricular dilation, and preserved left ventricular systolic function (EF 50-55%). The CMR study in 2018 confirmed stable scar morphology without extension.

In 2024, the patient presented to the emergency department with an electrical storm, characterized by multiple episodes of sustained VT, hemodynamic instability, and refractoriness to internal cardioversion via the S-ICD as well as pharmacological treatment. There were no factors promoting arrhythmia, such as electrolyte disturbances or ischemic events. Sedation was required, and the patient was placed on extracorporeal membrane oxygenation (ECMO) for circulatory support and continuous venovenous hemodiafiltration (CVVH) for renal support.

Given the failure of medical and device therapies, his case was urgently reviewed by a multidisciplinary heart team including cardiac surgeons, electrophysiologists, heart failure specialists, and intensivists. Based on prior electroanatomical mapping and imaging data, surgical resection of the arrhythmogenic aneurysm was deemed the only curative option.

**Figure 4:**
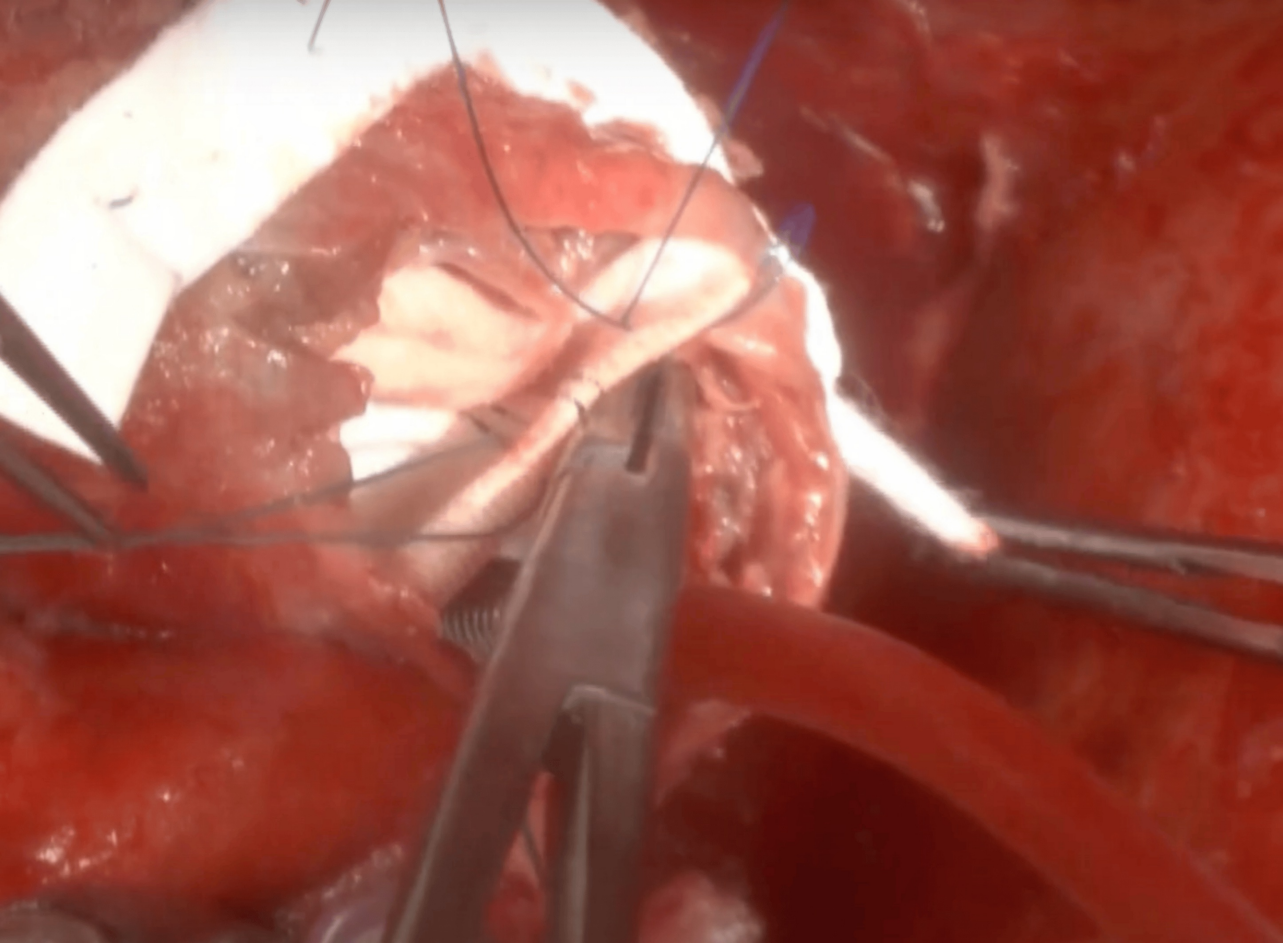
Intraoperative view during surgical ventricular reconstruction. The image shows the left ventricular cavity following ventriculotomy. The dyskinetic aneurysmal tissue has been resected, and a tailored circular Dacron patch is positioned for endoventricular plasty (Dor procedure).

The patient underwent surgery via median sternotomy. After instituting cardiopulmonary bypass and achieving cardiac arrest, a longitudinal left ventriculotomy exposed a dyskinetic aneurysm involving the posterobasal and posteroseptal segments. The aneurysmal tissue was carefully resected, and the left ventricle was reconstructed using a tailored Dacron patch according to the Dor procedure, restoring ventricular geometry and excluding the arrhythmogenic substrate (Figure 4). Intraoperative cryoablation was performed around fibrotic margins and within previously identified arrhythmogenic zones, applying a cryoprobe at −60 °C to −80 °C for 60 to 120 seconds per site, creating transmural lesions while preserving viable myocardium.

Postoperative recovery was uneventful. ECMO and CVVH were discontinued within 24 hours. He was discharged home one week later. He returned to work within two months, with full functional recovery.

**Figure 5:**
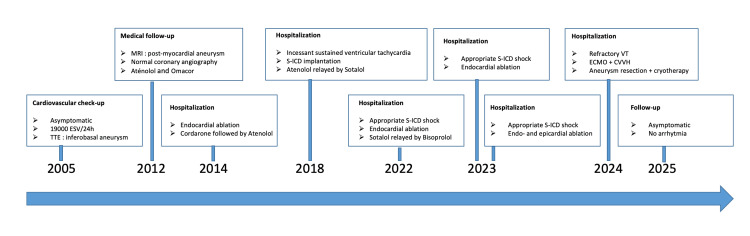
Timeline. Timeline depicting the patient’s medical history, including diagnostic tests, medications, and surgical procedures.

At the 11-month follow-up, the patient remained entirely asymptomatic. Intensive arrhythmia surveillance was conducted, including 48-hour Holter monitoring, ICD interrogations, and regular clinical evaluations. No sustained or non-sustained VT episodes were detected. Left ventricular EF remained stable (55%) on echocardiography. He remains completely asymptomatic, with no recurrence of ventricular arrhythmias on Holter monitoring and preserved left ventricular EF - an unprecedented outcome after nearly two decades marked by recurrent arrhythmic events (Figure 5).

## Discussion

Surgical resection of a ventricular aneurysm combined with cryoablation represents a highly selective therapeutic option for patients with refractory VT when standard medical therapy, implantable defibrillator therapy, and catheter ablation have all failed or are technically limited. Current guidelines support surgical ablation for refractory monomorphic VT (Class IIb recommendation), particularly when the arrhythmogenic substrate is inaccessible to percutaneous ablation approaches, such as in the setting of complex ventricular aneurysms [7].

However, most of the published data describing aneurysmectomy with cryoablation originates from patients with post-infarction aneurysms. These series report immediate electrical success rates of 80-90% and long-term survival free from VT recurrence or sudden cardiac death ranging from 60% to 85% at 5-14 years, with perioperative mortality between 1.9% and 9.5% [3,4,8]. While promising, these outcomes are not directly generalizable to post-myocarditic aneurysms, which differ in pathophysiology, scar morphology, and distribution. Unlike post-infarction aneurysms, which typically exhibit well-demarcated apical or anterior scars, post-myocarditic aneurysms often involve basal or lateral segments, with diffuse, patchy, or intramural scarring that complicates both mapping and surgical resection [8,10-13].

In this context, the Dor procedure - originally developed for ischemic aneurysm repair - has rarely been described for post-myocarditic substrates. Its use here reflects an expanding but still highly selective application beyond conventional ischemic indications. Careful preoperative imaging and electroanatomical mapping were essential in this case to confirm a localized, surgically accessible inferobasal scar. Although intraoperative electrophysiological mapping was not used during the procedure itself, prior invasive mapping from multiple failed ablations and detailed cardiac MRI data helped delineate target regions for surgical resection and cryoablation. This integration of imaging and prior electroanatomical data highlights the importance of a multidisciplinary strategy in such complex cases [14,15].

It is also important to recognize that this approach is not without risks. Surgical aneurysmectomy with cryoablation requires cardiopulmonary bypass, ventriculotomy, and patch reconstruction, with attendant risks of bleeding, ventricular dysfunction, arrhythmia recurrence, and perioperative mortality [3,4,6,14]. These factors limit its applicability to highly selected patients who have failed maximal pharmacologic therapy (including agents such as amiodarone, sotalol, and beta-blockers in this case) and advanced catheter-based strategies including high-density mapping, epicardial ablation, and repeated procedures [5,15]. While emerging techniques such as stereotactic body radiotherapy have shown promise for refractory VT, their long-term safety and effectiveness remain under evaluation, underscoring the need for individualized treatment planning [15,16].

This case is notable for several reasons. First, it demonstrates that even after nearly two decades of arrhythmia evolution, including multiple failed ablations and S-ICD shocks, surgical resection and cryoablation can achieve durable arrhythmia suppression when applied to a well-identified, anatomically localized substrate. Second, it underscores the critical role of thorough diagnostic workup and preoperative planning in carefully selecting patients who may benefit from such an invasive approach. Third, it adds to the limited literature documenting the feasibility and potential value of the Dor procedure combined with cryoablation specifically in post-myocarditic aneurysms, a setting with few formal guidelines and scarce data [9,15].

Finally, despite the excellent short-term result in this case, long-term surveillance remains essential given the patient's history of refractory VT. Continued device monitoring and imaging follow-up are warranted to detect possible arrhythmia recurrence or ventricular remodeling over time. Further research, including multicenter registries and prospective studies, is needed to better define patient selection criteria, technical strategies, and outcomes for surgical approaches to non-ischemic ventricular aneurysms.

## Conclusions

Surgical resection of a ventricular aneurysm combined with cryoablation, including the Dor procedure, represents a selective treatment option for refractory VT in highly chosen patients, especially when standard therapies, including antiarrhythmic medications, implantable cardioverter-defibrillators, and multiple catheter ablations, have failed. While this approach has demonstrated meaningful benefits in selected cases, its effectiveness and durability remain variable, supported primarily by limited case series data. It is therefore not universally applicable and must be weighed against the inherent surgical risks, including perioperative morbidity and mortality.

Importantly, while the Dor procedure is conventionally used for post-infarction aneurysms, this case illustrates its potential but still rare application in a post-myocarditic context, where scar morphology and substrate complexity pose distinct challenges. Careful patient selection, thorough preoperative imaging, integration of prior electroanatomical mapping, and precise intraoperative targeting are essential to optimize outcomes.
